# Application and Technical Challenges in Design, Cloning, and Transfer of Large DNA

**DOI:** 10.3390/bioengineering10121425

**Published:** 2023-12-15

**Authors:** Song Bai, Han Luo, Hanze Tong, Yi Wu

**Affiliations:** 1 Frontiers Science Center for Synthetic Biology and Key Laboratory of Systems Bioengineering (Ministry of Education), School of Chemical Engineering and Technology, Tianjin University, Tianjin 300072, China; 2 Frontiers Research Institute for Synthetic Biology, Tianjin University, Tianjin 300072, China

**Keywords:** large DNA, genome engineering, synthetic genomics, synthetic biology

## Abstract

In the field of synthetic biology, rapid advancements in DNA assembly and editing have made it possible to manipulate large DNA, even entire genomes. These advancements have facilitated the introduction of long metabolic pathways, the creation of large-scale disease models, and the design and assembly of synthetic mega-chromosomes. Generally, the introduction of large DNA in host cells encompasses three critical steps: design-cloning-transfer. This review provides a comprehensive overview of the three key steps involved in large DNA transfer to advance the field of synthetic genomics and large DNA engineering.

## 1. Introduction

As synthetic biology has advanced, the scale of genome design, assembly, and modification has expanded significantly. The field has transitioned from the early twentieth century’s focus on single-gene manipulation to encompass more extensive entities, including gene clusters, metabolic networks, and entire genomes. Advancements in genetics research have unveiled intricate and interconnected genetic information. When coupled with genome-scale genetic manipulation, this progress has greatly streamlined the transplantation of complex metabolic networks, eased the creation of large-scale disease models, and enabled the transplantation of entire genomes. These developments have had a profound and far-reaching impact on the fields of biomanufacturing, medicine, health, and gene therapy. The manipulation of small DNA has become mature and widely applied, but there are some inherent differences in properties between large and small DNA, such as being more fragile, more challenging to enter cells, and less stable once inside cells. This review primarily focuses on the manipulation of large DNA fragments or genomes ranging from hundreds of kilobases to megabases, a size range that often proves to be challenging.

**Figure 1:**
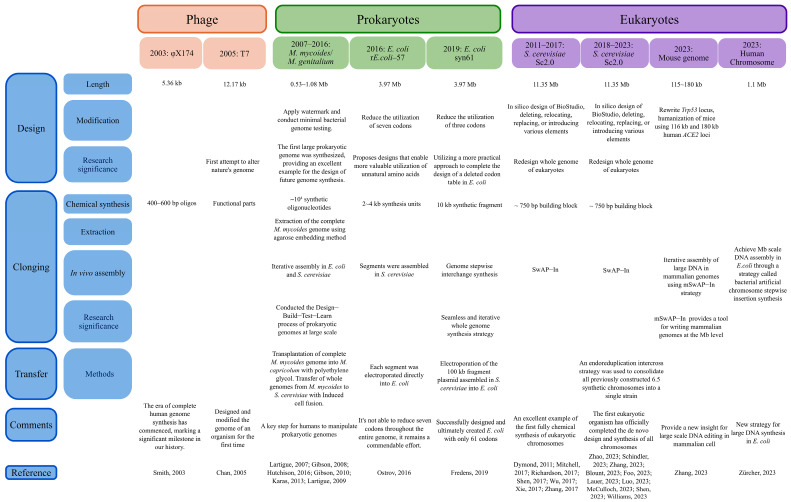
Classic cases in design, cloning, and transfer [1,2,3,4,5,6,7,8,9,13,14,15,16,17,18,19,20,21,22,23,24,25,26,27,28,29,30,31,32].

In 2007, J. Craig Venter provided a definitive description of genome transfer across species in a research-based article: “In this process, a whole bacterial genome from one species is transformed into another bacterial species, which results in new cells that have the genotype and phenotype of the input genome. The important distinguishing feature of transplantation is that the recipient genome is entirely replaced by the donor genome. There is no recombination between the incoming and outgoing chromosomes. The result is a clean change of one bacterial species into another” [1]. Large DNA transplantation can typically be categorized into three main phases: design, cloning, and transfer. Design refers to the deliberate modification, adjustment, or optimization of DNA with a specific purpose to achieve particular functions or properties. This process typically involves editing, deleting, relocating, replacing, or introducing various elements within genes, regulatory elements, and other functional components. Deleted elements include sequences and introns that are prone to genome instability. Relocated elements refer to changing the order of genes or gene clusters. Replaced elements involve genes encoding the same tRNA, substituted elements encompass codon substitutions and synonymous mutation substitutions. Introduced elements involve the incorporation of site-specific recombination systems. Large DNA can be acquired through in vitro cloning or in vivo assembly. In vitro extracting and cloning methods for large DNA include agarose embedding and Microcell-Mediated Chromosome Transfer (MMCT). There are already commercial kits available for extracting DNA at the hundred-kilobase (kb) level. The acquired large DNA can then be transferred using methods such as polyethylene glycol (PEG)-mediated transfer or electroporation. For in vivo assembly, the template may originate from DNA fragments synthesized and assembled in vitro before transferring, and these fragments are typically short segments from a few kilobases to several tens of kilobases. Alternatively, large DNA already present in the cells can be directly used for in vivo assembly through transfer methods such as cell fusion. In vivo assembly of large DNA has demonstrated success across various species, including *Mycoplasm*, *Saccharomyces cerevisiae* [2,3,4,5,6,7,8], *Escherichia coli* [9,10], and mammalian cells [11,12]. Currently, host cells commonly used for intracellular assembly include *E. coli*, *Bacillus subtilis,* and *S. cerevisiae*. Figure 1 summarizes the classic cases in design, cloning, and transfer.

The transfer of the large DNA is one of the core operations in genome manipulation. Manipulating large DNA presents more challenges compared with smaller ones due to its high molecular weight and susceptibility to shear forces, which can potentially diminish their efficiency of entry into cells. To surmount these challenges, numerous methods and strategies for transferring large DNA have been developed and applied across a range of cell types, including bacteria and fungi. Some of these techniques have also been explored for genome-level DNA transfers. The successful transfer of large DNA depends on factors including the integrity of the DNA, the efficiency of the transfer technique, and the stability of the transferred DNA within the recipient cell. Given these factors, transfer strategies tailored to address the vulnerabilities associated with large DNA are essential. After the transfer, the maintenance of transferred DNA in recipient cells can occur in two ways: integration into the genome or stabilization as an episome. This review provides a comprehensive overview of existing research on large DNA transplantation, elucidating the principles and limitations of various techniques, and offers a comprehensive and systematic summary while discussing the significant constraints involved.

## 2. Design of Large DNA

The advancement of synthetic genomics and the collaborative integration of multiple genomics approaches have enabled the rational design, synthesis, and assembly of genomes from scratch. This has also facilitated the design of their functions. The achievement of genome “editing” has opened up new avenues for gaining a deeper understanding of the fundamental processes of life [33]. In recent years, the development of synthetic genomics and the collaborative integration of various genomics approaches have made it possible to rationally design, synthesize, and reshape genomes. This has also led to the design of genomes to adapt them to a wide range of different organisms, including phages, bacteria, and fungi. Consequently, many studies have introduced additional design elements when assembling large DNA, such as customizing genes with specific functions (adding or deleting specific genes, modulating metabolic pathways), enhancing safety (removing pathogenic genes, increasing resistance to phage infections), and altering genome-scale (condensing or expanding genomes), among others.

In the context of genetic engineering, design involves the purposeful and strategic modification, fine-tuning, or enhancement of DNA to achieve a specific function or desired property. This tedious process often requires manipulation of genetic material through operations such as editing, deleting, relocating, replacing, or introducing various elements within genes, regulatory segments, and other key functional components. As the field of unnatural amino acids continues to grow, genomes can be designed to take advantage of unnatural amino acids by recoding stop codons. This technology has been successfully implemented in both *S. cerevisiae* and *E. coli*. When designing a genome, it is necessary to balance the expression of each gene in the original genome with the subsequent screening and application of the synthetic genome. This involves adding or modifying the coding of some genes and intergenic regions in the genome. At the same time, there are redundant sequences in the original genome of organisms, which can be deleted or relocated when synthesizing the genome to improve the compactness and efficiency of the genome. This process requires careful trade-offs to ensure that changes to the genome achieve their design goals without causing unintended negative effects.

Chan et al. [14] designed and synthesized the genome of phage T7, obtaining a semi-synthetic genome-encoded phage while establishing a simpler model of functional proto-manipulation. In 2008, the J. Craig Venter Institute (JCVI) accomplished the synthesis of the first complete *Mycoplasma* genome from scratch. During this process, the researchers employed antibiotic markers to disrupt the M408 gene to mitigate pathogenicity and utilized watermarks to identify the synthetic genome [15]. JCVI also reported a chemically fully synthesized 1.08 Mb *M. genitalium* genome and achieved transplantation of the synthesized genome to *M. capricolum*. Subsequently, JCVI embarked on the design and synthesis of the smallest bacterial genome ever created from scratch, known as JCVI-syn3.0. This synthetic genome is derived from *Mycoplasma* and has a size of only 5.3 Mb. The researchers pointed out that the process of minimizing the genome is essentially a quest for equilibrium between genome size and the organism’s growth state. They highlighted that apart from essential genes, a substantial number of genes related to promoting growth also constitute a crucial component of the minimal genome. The creation of JCVI-syn3.0, which serves as a research platform for investigating the fundamental functions of life, opens up new avenues for expanding our understanding of genome-wide design and exploration [16]. Among the prokaryotes, apart from *Mycoplasma*, the *E. coli* genome has been comprehensively engineered. Ostrov et al. [20] designed an *E. coli* genome that comprises only 57 codons. To reduce the utilization of seven codons (AGA, AGG, AGC, AGU, UUA, UUG, and UAG), the researchers systematically replaced 62,214 protein-coding regions with synonymous substitutions. They evaluated the functionality of 63 percent of these altered fragments and found that 91 percent of the essential genes were capable of performing their normal biological functions. In another notable achievement, a team led by Jason Chin at the MRC Laboratory of Molecular Biology succeeded in synthesizing the complete genome of *E. coli* from scratch. They also rewrote the 64 codons of the entire genome, reducing them to 61 codons. This resulted in the creation of a new strain named Syn61, which is governed by this synthetic genome [9]. Genome-wide codon substitutions can lead to the omission of the corresponding tRNAs (transfer RNAs), thereby establishing genetic isolation from other viruses, plasmids, or cells. This isolation can serve as a defense mechanism against specific viruses or reproductive isolation from particular cells, among other potential benefits.

In addition to genome synthesis efforts focused on improving and editing prokaryotic genomes, there have been numerous studies and noteworthy outcomes in genome modification endeavors for eukaryotes. *S. cerevisiae*, as the first fully sequenced eukaryotic organism, has been at the forefront of genome synthesis projects. The Sc 2.0 project, for instance, aims to chemically synthesize the complete genome of *S. cerevisiae* from scratch. This project is guided by three key principles: (1) the synthetic genome should be phenotypically similar or identical to wild-type yeast in terms of phenotype and growth; (2) the stability of the synthetic genome should be ensured; (3) the synthetic type of the genome should be genetically flexible for subsequent studies. The design of the Sc 2.0 project included the deletion of several elements: to improve genome stability, the researchers deleted several non-essential retrotransposons; the sub-telomeres of *S. cerevisiae* have two repetitive forms, Y’ and X. In the Sc 2.0 project, Y’ was completely deleted as it was found to have no known function. Additionally, the X form was uniformly replaced with a synthetic telomeric sequence that incorporated the conserved core X sequence. These modifications were made as part of the effort to streamline and optimize the yeast genome. In addition, approximately 285 genes in *S. cerevisiae* contain introns, and intron sequences lacking essential functions were targeted for deletion in the Sc 2.0 project. The Sc 2.0 project was designed in such a way that genes encoding the same tRNAs were reset. Indeed, in *S. cerevisiae*, 275 tRNA genes encode only 42 distinct tRNAs, resulting in redundancy. Additionally, tRNA genes are often accompanied by transposable elements, which can lead to chromosomal instability. To address this, the researchers in the Sc 2.0 project consolidated and relocated the tRNA sequences to a new synthetic chromosome. This restructuring helped streamline the genome and reduce potential sources of instability, contributing to the project’s goals in genome synthesis and optimization. The design of the Sc 2.0 project has made several substitutions to the component package, including the replacement of all stop codons TAG with TAA to facilitate subsequent expansion of the use of TAG for the synthesis of unnatural amino acids or reproductive isolation, etc.; synonymous mutations of a portion of all synthetic chromosome sequences, generally less than 10 codons in length, which will be designated “PCRTag” to distinguish synthetic chromosomes from wild-type ones; synonymous substitution of portions of sequences to eliminate specific enzymatic site. The Sc 2.0 project incorporated a significant innovation by inserting symmetrical loxP sequences into the 3′ non-transcribed regions of all non-essential genes, as well as into synthetic sequences where deletions had been made. This introduction of loxP sequences established a rapid evolutionary rearrangement system at the global level of the genome, which was aptly named SCRaMbLE (synthetic chromosome rearrangement and modification by loxP-mediated evolution). With these enhancements, the size of the synthetic *S. cerevisiae* genome synthesized by the Sc 2.0 project was reduced by 8%, and nearly 1.1 Mb of the sequence was deleted, inserted, or modified. It is worth noting that the synthetic yeast genome design for the Sc 2.0 project was facilitated with the assistance of computers, utilizing the BioStudio software developed by the Joel Bader team at Johns Hopkins University. BioStudio is an open-source framework specifically designed for the assembly of eukaryotic genomes. It employs a series of Perl scripts to achieve hierarchical assembly of nucleotides into genome-scale designs, enabling systematic tracking of the genome assembly process through design, debugging, and modification phases [4].

**Figure 2:**
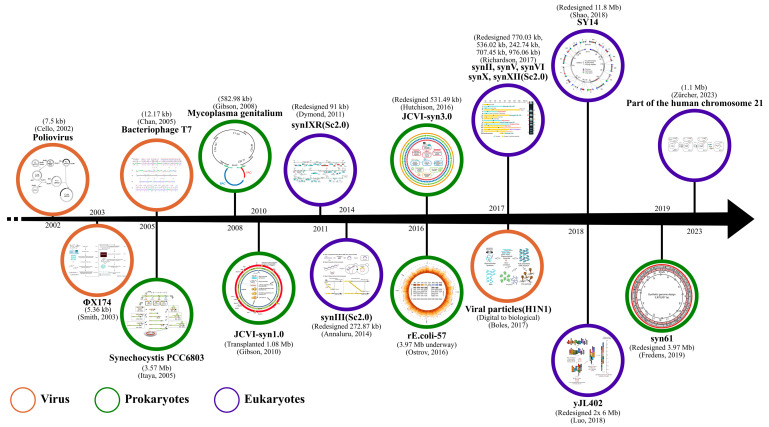
Milestones in large DNA engineering. The manipulation of large DNA is a challenging task. Over time, researchers have progressively embarked on exploring smaller-scale viral genomes and subsequently attempted to manipulate larger prokaryotic and eukaryotic genomes [2,4,9,13,14,15,16,17,20,32,34,35,36,37,38,39].

In 2017, five separate and independent studies were published in the journal Science, reporting significant progress in the field of synthetic genomics related to *S. cerevisiae*. These studies focused on the synthetic genomes of different yeast strains, including synII, synV, synVI, synX, and synXII. Shen et al. [5] completed the synthesis of *S. cerevisiae* chromosome II, which has a length of 770,035 bp. They comprehensively characterized this synthetic chromosome using multi-omics approaches. Xie et al. [7] perfectly synthesized *S. cerevisiae* chromosome V and constructed a cyclic form of synV derivatives, which had slightly lower sporulation viability under various assay conditions, and the rest of the features were not significantly different from those of the wild-type chromosome. Mitchell et al. [3] reported work on the synXVI chromosome with a length of 242,745 bp and provided an in-depth analysis of the problem of growth defects brought about by it, and the study identified defects in mitochondrial function brought about by synonymous mutations in the *PRE4* gene, as well as interference with HIS2 transcription caused by deleted tRNAs and the introduction of the loxPsym locus. Zhang et al. [8] synthesized the synXII chromosome using a strategy called Meiotic Recombination-mediated Assembly (MRA) and pioneered the replacement of the original sequence with the rDNA sequence of *S. bayanus* so that it would be impossible to distinguish whether it was *S. cerevisiae* or *S. bayanus*. Wu et al. [6] developed a high-throughput strategy called pooled PCRTag mapping (PoPM) for identifying accidental errors during synthetic chromosome assembly, and a technique for efficiently repairing chromosome duplications and rearrangements of large chromosome segments using meiotic homologous recombination. It is worth mentioning that all the above studies introduced the SCRaMbLE system during the synthetic genome process to achieve a rapid evolutionary drive of the genome. In addition to the Sc 2.0 project, Luo et al. [34] successfully fused yeast chromosomes together using CRISPR-Cas9 technology to create a series of strains with progressively fewer chromosomes. A strain carrying only two chromosomes of approximately 6 Mb in size showed slight transcriptional changes and no apparent growth defects. Shao et al. [35] constructed single-chromosome yeast by sequential end-to-end chromosome fusion and mitotic deletion. The fusion of 16 naturally occurring linear chromosomes into a single chromosome led to a significant change in the global 3D structure of the chromosomes. However, the single-chromosome yeast and the wild-type yeast have nearly identical transcriptomes and similar phenotypic characteristics, but the strain exhibits reduced growth, competitiveness, gamete production, and viability. Figure 2 summarizes the milestones in large DNA engineering.

## 3. Cloning of Large DNA

Cloning of large DNA can be categorized into two main approaches: in vitro synthesis/cloning and in vivo assembly. In vitro synthesis refers to the process of synthesizing oligos DNA fragments from scratch and then assembling the oligos into larger fragments through in vitro cloning. In vitro cloning can use fragments synthesized in vitro or extracted from organisms directly. Large DNA templates can be extracted by disrupting various biological structures, such as the cell wall, cell membrane, and nuclear membrane, using a combination of physical and chemical techniques. It is worth noting that large DNA molecules have significantly greater molecular weight compared with small DNA, which makes in vitro manipulation challenging due to their increased susceptibility to shear forces. Currently, common methods for in vitro cloning large DNA include agarose embedding and microcell-mediated chromosome transfer (MMCT). Additionally, some commercial kits utilize principles like alcohol precipitation, anion exchange, and magnetic bead adsorption to extract large DNA, with the capacity to handle DNA fragments of up to 100 kb in size.

### 3.1. In Vitro Synthesis/Cloning of Large DNA

The chemical synthesis technique for synthesizing oligonucleotides from scratch has been developed for over 40 years. However, accurately synthesizing oligonucleotides exceeding 200 nucleotides from scratch remains challenging [40,41]. The use of DNA polymerase, specifically terminal deoxynucleotidyl transferase (TdT), has the potential to accurately polymerize longer oligonucleotides, but obstacles remain in the commercialization process due to the necessity of adding high-purity dNTPs one at a time [42]. To obtain longer fragments, further assembly of synthesized oligonucleotides is required. Polymerase chain assembly (PCA) can be employed to polymerize single-stranded oligonucleotides into double-stranded DNA segments. Double-stranded DNA segments can then be assembled into longer fragments using polymerase chain reaction (PCR), sequence and ligation independent cloning (SLIC), Golden Gate, or Gibson assembly. Using these methods, it is possible to obtain several kilobases of DNA [43]. Currently, it is not feasible to synthesize large DNA in the range of several hundred kilobases to megabases in vitro directly. The DNA fragments assembled in vitro can be utilized as templates for in vivo assembly, enabling assembly at larger size scales [44]. While the cost of in vitro synthesis is relatively high, it remains the only method currently available for obtaining de novo synthesized DNA fragments. When dealing with genomes that require extensive modifications on a large scale or genomes that need to be designed from scratch, in vitro synthesis remains an unavoidable step. For example, in the Sc 2.0 project, the design of minichunks allows for the assembly of long segments through a ‘one-pot’ in vitro assembly. Ultimately, this enables the integration of tRNA onto a single chromosome [8]. In the process of simplifying the *E. coli* codons, during the in vitro synthesis of short segments, TCG, TCA, and TAG were respectively replaced with AGC, AGT, and TAA. This ultimately resulted in the synthesis of the *E. coli* genome with 61 codons [9].

When using DNA extracted from organisms as a template for in vivo assembly, the shearing forces during the extraction process often lead to damage in large DNA fragments. The agarose embedding method can alleviate this issue. First, cells are embedded in an agarose gel matrix. Subsequently, the cell membrane, nuclear membrane, and other biological membranes are disrupted using cell lysate, allowing proteins, RNA, and other cellular components to enter the agarose gel and diffuse into the lysate. During this process, the DNA remains entangled within the cell’s nuclear scaffold and is not exposed to external factors such as shear forces. This ensures the preservation of DNA integrity. Subsequent separation and purification using pulsed-field gel electrophoresis can yield high-quality large DNA suitable for transfer. The agarose embedding method has been successfully employed to extract large DNA at the Mb level from various organisms, including fungi [45,46,47], plants [48,49], and mammalian cells [50,51,52]. Lartigue et al. [1] utilized the agarose embedding technique to extract and isolate the 1.1 Mb-sized genome of *M. mycoides*. Subsequently, they transferred this genome to *M. capricolum* using PEG-mediated methods, with a transformation efficiency ranging from 2.5 × 10^2^ to 7.5 × 10^2^ colonies/μg DNA. Lee et al. [45] employed the agarose embedding technique to isolate and purify a 2.3 Mb-sized Yeast Artificial Chromosome (YAC). They took measures to neutralize the negative charge of the DNA using substances such as poly-L-lysine or polyethyleneimine, resulting in a more compact DNA molecule. This approach prevented the genome from being exposed to shear forces. Ultimately, they successfully transfected the YAC into mouse embryonic stem cells using liposome transfection methods. The agarose embedding method effectively shields large DNA from external shear forces during the cloning process, thereby ensuring its integrity. After cloning, methods for introducing DNA into recipient cells include PEG-mediated protoplast transformation, microinjection, and lipofection, among others. In theory, the agarose embedding method has a wide range of applications and is not limited by donor and recipient cell types. However, currently, while it is feasible to extract DNA at the megabase (Mb) level using this method, it remains relatively inefficient.

MMCT is a technique used to transfer exogenous chromosomes into recipient cells by creating microcells. The MMCT process can be divided into three key steps: nucleation, nuclear removal, and cell fusion [53]. Fusion methods for MMCT encompass PEG, electrofusion, and virus-mediated approaches, followed by screening to isolate positive clones. Among these, PEG and electrofusion methods are associated with high cytotoxicity, while virus-mediated MMCT is the more commonly employed technique. MMCT finds extensive application in animal disease models and the preparation and construction of humanized mouse antibody sequences. Kazuki et al. [54] achieved complete correction of genetic defects by transplanting human artificial chromosomes (HACs) containing complete genomic anti-dystrophy protein sequences into induced pluripotent stem cells using the MMCT technique. Kuroiwa et al. [55] constructed mEScs containing the 10 Mb region of HAC on human chromosome 22 using MMCT. They subsequently employed these cells to generate chimeric mice. Currently, MMCT is widely utilized for extracting chromosomes from mammalian cells. This method is currently the only reported successful approach for extracting DNA at the natural chromosomal scale in mammals. Throughout the process, the DNA is consistently protected by the cell membrane, avoiding exposure to shear forces. However, the application of MMCT is limited to mammalian cells, and donor cells are restricted to those capable of producing microcells (DT40, A9, CHO, and HT1080), resulting in lower universality.

**Figure 3:**
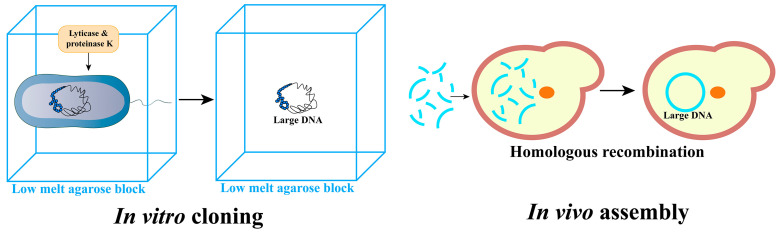
Schematic diagram of in vitro cloning and in vivo assembly. Obtaining intact genome within agarose gel blocks. Assembling large DNA utilizing homologous recombination.

Both the agarose embedding method and MMCT are capable of cloning complete genomes or chromosomes at the Mb level but are considered cumbersome and inefficient for studies that require large DNA sizes at the hundred kb level. In response to this, several simplified extraction methods have been developed and extended into commercial kits. These methods include alcohol precipitation, anion exchange columns, and magnetic bead adsorption. Alcohol precipitation involves using isopropanol to reduce the solubility of large DNA in the aqueous phase, causing it to precipitate. Similarly, the use of organic reagents, such as chloroform phenol extraction of DNA, operates on the same solubility principles for genomic DNA extraction. These methods provide more streamlined and efficient options for obtaining large DNA fragments in the range of hundreds of kb. The anion-exchange column method refers to the interaction between negatively charged phosphate groups in DNA and positively charged molecules on the exchange column. In low salt conditions, the DNA binds to the column, while impurities are washed away because they cannot bind to the column. The DNA is ultimately obtained by elution with a high salt buffer. Anion exchange columns have been fully commercialized, making them readily accessible for laboratory use. Wang et al. [56] employed Qiagen Blood & Cell Culture DNA Kits to extract genomic DNA from various sources, including mouse melanoma B16 cells, human embryonic kidney 293T cells, and human blood, to clone heterologous genomes. They successfully cloned a complete DNA fragment of approximately 50 kb using this method. Noyes et al. [57] on the other hand, utilized the QIAGEN MagAttract HMW DNA kit to isolate DNA fragments larger than 80 kb. The high-quality DNA obtained from this isolation process was suitable for 10× Genomics linked-read sequencing. Figure 3 illustrates the in vitro cloning and in vivo assembly of large DNA.

### 3.2. In Vivo Assembly of Large DNA

In vivo assembly, when combined with cell fusion, can circumvent the shear forces that impact large DNA during in vitro cloning, thereby ensuring the integrity and quality of the resulting large DNA fragments. Typically, in vivo assembly makes use of model organisms as hosts, including *E. coli*, *B. subtilis*, and *S. cerevisiae*. *E. coli*, in particular, provides advantages such as straightforward culture conditions and rapid amplification, leading to a notable reduction in the assembly cycle. With the incorporation of its λRed homologous recombination system, it becomes feasible to integrate multiple tens of kb-sized fragments into expression vectors or genomes [58]. Jérôme et al. [32] developed a method for in vivo assembly of Mb-scale genomes in *E. coli* called bacterial artificial chromosome stepwise insertion synthesis (BASIS). They applied BASIS to assemble a 1.1 Mb human genome and concurrently developed a continuous genome synthesis method that involves the successive replacement of 100 kb segments of the *E. coli* genome with synthetic DNA. Using this continuous genome synthesis approach, they successfully synthesized a 0.5 Mb *E. coli* genome. The RecA system of *B. subtilis* has strong homologous recombination abilities. *B. subtilis* possesses a natural ability to uptake exogenous genetic material and exhibits a robust competence for horizontal gene transfer, making it relatively adept at transferring DNA to other prokaryotes. Additionally, the genome of *B. subtilis* can serve as a direct carrier for incorporating exogenous DNA [37].

*S. cerevisiae* naturally possesses a robust homologous recombination capacity and stands as one of the most commonly employed host cells in the field of genome synthesis. The efficiency of homologous recombination in *S. cerevisiae* is influenced by several factors, including the number and length of DNA fragments involved, as well as the length of homologous sequences. In general, there tends to be a negative correlation between assembly efficiency and the quantity as well as the length of fragments that need to be assembled. Furthermore, with regard to the length of homologous sequences, Sugawara et al. [59] found that recombination induced by double-strand breaks necessitates a length of 63–89 base pairs. Simultaneously, an increase in the length of homologous sequences enhances the assembly efficiency. Genome assembly methods facilitated by *S. cerevisiae* homologous recombination are widely employed due to their capability to directly assemble multiple DNA fragments into complete plasmids or integrate them into the genome. Gibson et al. [15] successfully assembled the *M. genitalium* genome JCVI-1.0 of approximately 583 kb in size using the homologous recombination system of *S. cerevisiae*. This accomplishment followed the in vitro assembly of six 100 kb fragments. The international “Synthetic Yeast Genome Project” has the objective of designing 16 chromosomes for *S. cerevisiae*. As of now, the project has completed the design and construction of 6.5 synthetic chromosomes in *S. cerevisiae* [2,3,4,5,6,7,8]. Li et al. [12] employed the MRA approach to successfully assemble 1.1 Mb and 700 kb human TCRαβ motifs using *S. cerevisiae*. These large circular DNA molecules were introduced into *S. cerevisiae* via protoplast fusion and then cleaved by Cas9 to generate linear DNA fragments for assembly using the homologous recombination system. Currently, our team has developed a CRISPR-Cas9-mediated haploidization method that bypasses the natural meiosis process in yeast. Building on this, we have further devised a simple and efficient genome assembly method designated HAnDy (Haploidization-based DNA Assembly and Delivery in yeast). This method allows for the efficient assembly and transfer of Mb-scale large DNA fragments. We have successfully assembled a synthetic chromosome of 1.024 Mb, containing 542 exogenous genes, using HAnDy. Furthermore, this synthetic chromosome was directly transferred into six different yeast strains (Cell Research, under revision). Additionally, studies conducted by Luo et al. [34] and Shao et al. [35] involved the construction of two 6 Mb chromosomes or one 12 Mb chromosome in *S. cerevisiae*, illustrating the remarkable chromosome-carrying capacity of this organism. This suggests that the current state of research is far from reaching the assembly limits of *S. cerevisiae*. Compared with prokaryotes, the genome-scale of the yeast *S. cerevisiae* has inherent advantages, featuring greater assembly potential. Genomes prepared in vivo can either be manipulated after treatment with agarose packages or directly converted into protoplasts for fusion-mediated genome transfer [1,18,60]. Compared with in vitro cloning, in vivo cloning is typically more time-consuming, as it involves waiting for cells to complete transformation and growth. Although the MMCT method can achieve the transfer of DNA of natural chromosome size, accomplishing gene modifications on such a large scale still requires the combination of in vivo cloning. Moreover, in cloning, assembling, or transferring large DNA, CRISPR tools have significantly enhanced the efficiency of genome editing and assembly. This has further propelled the development and innovation of numerous advanced technologies in the field.

## 4. Transfer of Large DNA

Following the cloning of large DNA, the transfer process assumes a crucial role within the domain of large DNA manipulation. In this chapter, we will introduce the large DNA transfer methods applicable to different categories of recipient cells including fungi and bacterial cells, respectively.

### 4.1. Transfer Methods of Large DNA for Fungi as Recipient Cells

**Figure 4:**
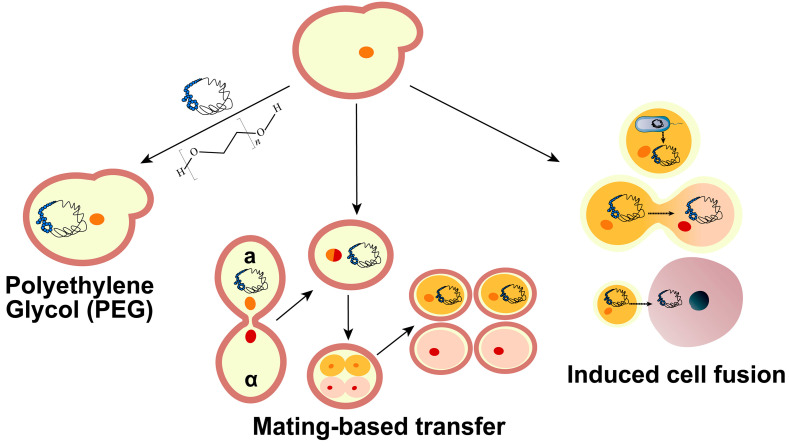
Schematic diagram of the large DNA transfer methods for yeast as recipient cells. PEG enhances microbial cell wall permeability. This facilitates DNA adsorption onto cell membranes to complete the transformation. Yeast of a and α mating types, through the process of mating, generate four spores, allowing for the transfer of large DNA during this process. Induced cell fusion can occur among bacteria, yeast, and mammalian cells.

When fungi are used as recipient cells, in the case of yeast, for example, large DNA transfer can be achieved using PEG-mediated transformation, electroporation, induced fusion, and yeast mating (Figure 4).

PEG is a widely employed polymer in biological manipulations for facilitating the transformation of microorganisms possessing cell walls, such as *E. coli*, yeast, and *Aspergillus*. In the PEG-mediated transformation, PEG chains can bind to the exposed sugar chains on the cell membrane surface through hydrogen bonding. This enables cells to adsorb DNA molecules, which are initially attached to the cell surface and are subsequently stimulated to enter the cell through thermal processes, thereby completing the transformation [61]. *S. cerevisiae* possesses a highly efficient homologous recombination system, enabling the one-step assembly of large fragments following the co-transformation of multiple fragments. Postma et al. [62] co-transformed 44 fragments into yeast cells using PEG-mediated transformation and assembled them in one step into a 100 kb plasmid, with a transformation efficiency ranging from 10^−3^ to 10^−5^ colonies/cells. In the Sc 2.0 project, co-transformation was used to assemble smaller minichunks into 30–60 kb fragments, allowing the replacement of corresponding segments of the wild chromosome [2,3,4,5,6,7,8]. To further enhance transformation efficiency and increase the upper limit of the transformation scale, yeast cells can be treated as protoplasts, thus eliminating the impediment posed by the cell wall to material uptake [63]. Gibson et al. [17] transformed multiple 100 kb fragments into *S. cerevisiae* by protoplast transformation during the construction of JCVI-syn1.0 and successfully assembled a 1.08 Mb synthetic genome.

Electroporation is a widely employed transformation method for both microbial and mammalian cells. During electroporation of yeast, the yeast cell wall is typically pretreated using chemical or biological methods to enhance its permeability, which transformation efficiency can reach up to 10^2^–10^5^ colonies/µg DNA [64]. Following this pre-treatment, short but potent electrical pulses are externally applied to the cell using an electroporation device. These pulses induce a rapid loss of the cell membrane’s semipermeable properties, thus facilitating the entry of exogenous substances into the cell [65]. Similar to PEG-mediated transformation, treating cells as protoplasts can also improve the efficiency and upper limit of electroporation, enabling the transformation of 10 kb of DNA in *S. cerevisiae* [66].

Cell fusion, a versatile category of large DNA transfer methods, can involve yeast as both the donor and recipient. Compared with PEG-mediated transfer and electroporation, cell fusion-mediated transfer avoids shear force damage to DNA during the transfer process. Cell fusion can be categorized into spontaneous and induced fusion. Spontaneous fusion occurs naturally between a donor and recipient, as seen in the mating process of *S. cerevisiae*. In the Sc 2.0 project, researchers employed multiple rounds of yeast mating-mediated transfer to accomplish the assembly of multiple synthetic chromosomes [3,8,38,67]. The main challenge in integrating multiple synthetic chromosomes into the same yeast strain is the uncontrolled recombination that can happen between synthetic and wild-type chromosomes during meiosis. To address this challenge, various methods have been developed. One approach involves inserting the Gal promoter near the chromosome centromere to create conditional centromeres, as employed by Richardson et al. [4]. Another strategy, utilized by Zhou et al. [67], employs the Vika/vox system to knock out wild-type chromosomes that correspond to synthetic chromosomes in yeast. Xu et al. [68] have developed a CRISPR-Cas9 chromosome driver system capable of deleting entire chromosomes by targeting the centromere region. Additionally, Guo et al. [69] utilized an abortive mating method that hinders nuclear fusion, facilitating chromosome transfer between synthetic and wild-type yeasts. This approach, combined with CRISPR-Cas9 chromosome elimination, streamlines the process by avoiding traditional mating complexities like sporulation and spore disassembly.

Induced cell fusion refers to the use of inducers (e.g., PEG, electrical stimulation, viral proteins, etc.) to promote fusion between two cells. Induced fusion can occur between cells of the same species, or it can break the barrier of reproductive isolation and occur between cells of different species. Zhou et al. [70] accomplished the assembly of synthetic genomes over 1 Mb within *S. cerevisiae* by transferring large-scale circular DNA into recipient cells through fusion and subsequently releasing linear fragments using the Cas9. Yeast also can acquire large-scale DNA through fusion with cells of other species. For instance, Karas et al. [18] achieved the successful transfer of the 1.8 Mb genome of *M. mycoides* into *S. cerevisiae* through cell fusion. Additionally, Ruiz et al. [63] accomplished the transfer of the *Mycoplasma* genome into *S. cerevisiae* by combining fusion-mediated transfer with the CReasPy-Cloning technique, which involved inserting yeast elements into the bacterial genome. Compared with mating, the induction of cell fusion is relatively more complex and less efficient, but it can overcome reproductive barriers between species to accomplish DNA transfer.

In addition to the aforementioned methods, *Agrobacterium*-mediated transformation can also be employed for the transformation of fungi. While our review primarily focuses on fungal receptors with DNA transfer sizes exceeding 100 kb, and the maximum reported transfer from Agrobacterium to fungi is 75 kb [71], considering Agrobacterium’s application in plant transformation exceeding 100 kb [72], we believe it holds the potential for a larger payload capacity.

*S. cerevisiae* possesses an efficient homologous recombination system and is frequently employed for the direct integration of transferred large DNA. As an example, in the Sc 2.0 project, the assembly of complete chromosomes relies on sequential SwAP-In (switching auxotrophies progressively for integration), a method rooted in the homologous recombination capabilities of *S. cerevisiae* [2,3,4,5,6,7,8,73]. Furthermore, in yeast, YACs can be employed to maintain the episomal stability of large DNA fragments. YACs are available in both cyclic and linear forms, with linear YACs requiring the inclusion of an auto-replicative sequence, a centromere, and a telomere to maintain their linear structure. YACs have a carrying capacity of up to 2.5 Mb, far exceeding that of conventional plasmids [74]. Based on the basic core elements of YACs, studies have successfully merged 16 chromosomes of *S. cerevisiae* into a single chromosome of 12 Mb [34,35]. These studies show that there is still potential for expansion of the carrying capacity of YACs. Table 1 summarizes the transfer methods for fungi and bacteria, respectively.

### 4.2. Transfer Methods of Large DNA for Bacterial as Recipient Cells

**Figure 5:**
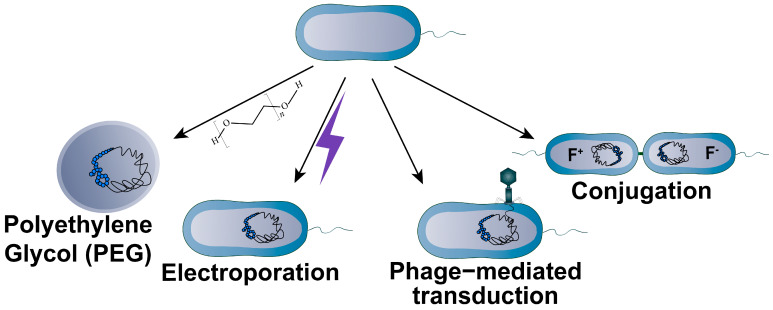
Schematic diagram of the large DNA transfer methods for bacterial recipient cells. Bacterial transformation mediated by PEG involves the mutual adsorption of exogenous DNA and competent cells, while electroporation utilizes an electric field to enhance cell membrane permeability, allowing DNA to enter the cells. Conjugation transfer relies on the T4SS to facilitate horizontal gene transfer of plasmids or ICEs, whereas phage transduction depends on phages infecting bacteria to deliver exogenous DNA to recipient cells.

Commonly employed gene transfer methods for bacteria include PEG-mediated transformation, electroporation, conjugation, and phage transduction (Figure 5). For PEG-mediated transformation, some bacterial strains serve as natural receptor cells capable of efficiently accepting exogenous substances, while others may require pretreatment with specific reagents, such as triazole buffer or divalent metal ions, to create a receptive state that can then be transformed using PEG. For example, Lartigue et al. [19] extracted 1.1 Mb genome of *M. mycoides* using agarose embedding and subsequently transformed it into *M. capricolum* by PEG-mediated transformation. Electroporation is another frequently employed method for introducing large DNA into bacteria. The electroporation enables the transformation of bacterial artificial chromosomes (BAC) as long as 120 kb into *E. coli* with an efficiency of 7 × 10^8^ colonies/μg DNA, and it has proven to be a valuable tool in genetic engineering [75]. Conjugation and phage transduction are transfer methods that rely on specific biological processes. Conjugation is a horizontal gene transfer mechanism that depends on the bacterial type IV secretion system (T4SS), which allows for the transfer of removable DNA on the splice transfer plasmid or integrally conjugated elements (ICEs) embedded in the chromosome into the recipient cell. Isaacs et al. [76] remodeled standardized *E. coli* codons and successfully transferred half the genome to recipient cells by conjugation. Based on the conjugation elements of *B. subtilis* (ICEBs1), Brophy et al. [77] designed a controlled conjugation system and demonstrated that it could efficiently transfer DNA in at least 35 g-positive strains, and its transformation efficiency ranged from 10^−1^–10^−7^ colonies/cells. Phage transduction is another major horizontal gene transfer mechanism in bacteria. The P1 phage cloning system developed by Sternberg et al. [78] is capable of transferring 95–100 kb of DNA to *E. coli*.

For bacteria, tools used for the post-transfer stability of large DNA fragments include BAC and homologous recombination. BACs are circular artificial chromosomes based on bacterial fertility factors (F factors) with a carrying and stability capacity of up to 350 kb [79]. BAC replication begins at replication start S (oriS) and is tightly regulated by the *repE* and *repF* gene products encoded by the F factor, which maintains a low copy number in *E. coli* [80]. Certain bacteria, such as *B. subtilis* and *Clostridium acetobutylicum*, possess robust endogenous homologous recombination systems. Using *B. subtilis*’ homologous recombination system, Itaya et al. [37] successfully created a composite genome of 7.7 Mb by combining the 3.5 Mb *Synechocystis* genome with the *B. subtilis* genome. For bacterial cells lacking efficient endogenous homologous recombination systems, exogenous homologous recombination systems can be introduced as tools for genetic manipulation. The Red protein from the λ phage can facilitate homologous recombination of DNA fragments with short homology arms (~50 bp). Isaacs et al. [76] achieved the substitution of a 2.3 Mb chromosome fragment using the λ Red system in their work on remodeling standardized *E. coli* codons.

## 5. Barriers of Large DNA Transplantation

During the process of transplanting large DNA, the efficiency of transfer is influenced not only by the cloning of the large DNA and the method of transfer and stabilization but also by the inherent characteristics of both donor and recipient cells. These characteristics encompass cell structure and the natural defense mechanisms existing within the cell. Cellular components such as cell walls, cell membranes, and nuclear membranes act as natural barriers to prevent the ingress of foreign genetic material into the cell. Meanwhile, the intracellular defense systems vary depending on the cell type, with systems like CRISPR, widely distributed in bacteria and archaea [81], and restriction-modification (R-M) systems in bacteria [82] playing significant roles.

Cell walls are prevalent in a variety of organisms, including plants, bacteria, fungi, algae, and certain prokaryotes. However, notably, *Mycoplasma spp.* lack cell walls. These microorganisms’ cell walls primarily consist of polysaccharides, complemented by various proteins and lipids, rendering them robust and supportive. They play a crucial role in maintaining the cell’s structural integrity and shielding it from external influences. The cell wall serves as the primary defense against the intrusion of exogenous genetic material. Consequently, for many microorganisms possessing cell walls, the initial transformation step involves either the removal or weakening of these cell walls. In some bacterial species, achieving a receptive state amenable to transformation while preserving cell wall integrity can be attained during their logarithmic growth phase by subjecting them to multiple washes with a hypertonic solution. Conversely, in the case of fungi or algae, the selection of specific enzymes targeting their cell wall components can render their cells susceptible to transformation [83]. For non-model strains or cells characterized by thick cell walls, a combination of electrical stimulation or chemical treatments may be necessary to facilitate successful transformation [84].

The cell membrane, also referred to as the plasma membrane, exhibits distinct characteristics depending on whether the cell possesses a cell wall or not. In cells with cell walls, it is situated between the cell wall and the cell’s interior, whereas in wall-less cells, it constitutes the outermost layer. Comprising lipids, sterols, and proteins, the cell membrane forms a semi-permeable, fluidic structure. Compared with the cell wall, the cell membrane offers considerably less resistance to exogenous genetic material, enabling the cell to readily assume a receptive state. Eukaryotic microorganisms feature a unique cellular structure known as the nuclear membrane. This membrane comprises two layers, the inner and outer unit membranes, and is adorned with numerous nuclear pores that facilitate substance exchange between the nucleus and cytoplasm. These pores also serve as gateways for the entry of exogenous genetic material into the nucleus. However, the capacity of chromosome-scale genetic material to traverse the nuclear pores into the nucleus remains an unexplored area of research. In contrast, bacteria lack a nuclear membrane, rendering them naturally adept at receiving significant amounts of exogenous genetic material. The JCVI team has extensively reported on chromosome transplantation in *Mycoplasma*, an organism devoid of both cell wall and nuclear membrane, significantly simplifying the experimental process. For instance, Brown et al. [60] successfully assembled a 1.12 Mb yeast artificial chromosome in *S. cerevisiae* and transferred it from yeast to mammalian cells through cell fusion. In this procedure, the efficiency of intercellular delivery was boosted approximately tenfold by synchronizing mitosis in mammalian cells. Additionally, the experimental design enhanced vector delivery efficiency by 300-fold. Consequently, many semi-open or open mitotic eukaryotes could employ a similar experimental strategy to enhance the success of large-scale exogenous genetic material transformation. However, for certain eukaryotes like budding yeast, mitosis occurs within an entirely enclosed process, and the nuclear membrane remains intact throughout [85]. As a result, extending mitosis, as previously performed, is not feasible for large-scale genetic material transformation operations in these organisms.

In addition to the cellular structure, the cell’s intrinsic biological defense system constitutes a highly precise and formidable barrier to genetic transformation. CRISPR is a natural immune system found in prokaryotic cells. When these cells encounter specific viral invasions, they can capture and integrate genes from the invading viruses into their DNA sequences. Upon subsequent viral encounters, the cell can recognize these stored DNA sequences and direct the Cas9 enzyme to cleave the viral DNA, effectively defending against the invasion. Leveraging this recognition and defense mechanism, researchers have transformed CRISPR-Cas9 into a precise and convenient genetic editing tool [86]. CRISPR exists in a few prokaryotes and archaea, although CRISPR-like systems have recently been discovered in eukaryotes: the RNA-guided DNA-cleaving enzyme, Fanzor [87]. Nevertheless, the CRISPR system does not typically serve as a barrier to genetic transformation in many model and non-model strains. The R-M system is the more common and influential intracellular defense system for genetic transformation than CRISPR. The R-M system is a system present in bacteria that protects the cell from exogenous DNA and consists mainly of restriction endonucleases and methylases. The former recognizes specific cleavage sites and cleaves exogenous DNA, while the latter can methylate its own DNA sequence-identical cleavage sites as a means of escaping damage by restriction endonucleases. In some R-M systems, the two are distinct proteins, while in some systems they are one large restriction-modification complex enzyme. Genetic transformation operations have been studied by in vitro methylation of the genetic material to be transferred or by knocking out the R-M system of the target strain [19].

## 6. Concluding Remarks

With the increasing manipulation of gene sizes, the field of synthetic genomics is rapidly advancing. Genome synthesis has offered us a profound new perspective for comprehending living systems. Subsequent genome writing efforts have sparked investigations into various biological phenomena across a wide array of organisms. Synthetic genomics has played an indispensable role in the field of human health. The development of design-cloning-transfer for large DNA has provided effective tools for progress in areas such as the transplantation of complex metabolic pathways, modeling of multi-gene complex diseases, and the development of vaccines involving larger gene scales. For example, during the COVID-19 pandemic, the generation of the SARS-CoV-2 genome has the potential to promote the unraveling of disease mechanisms and the development of vaccines [88]. Zhang et al. [31] constructed genetically engineered mouse models by introducing 116 kb and 180 kb humanized *ACE2* loci in *S. cerevisiae*, which, when compared to the existing *K18-hACE2* models, presented milder symptoms upon exposure to SARS-CoV-2 and more closely resembled human infection models.

Currently, the synthesis of genomes is mostly restricted to model organisms with user-friendly genetic tools and mature manipulation techniques, such as *S. cerevisiae*, *E. coli*, *B. subtilis*. The design steps rely on an understanding of gene structure and function, and as the DNA scale increases, more complex gene interactions need to be taken into consideration. In the synthesis of genomes at the Mb level, the design of excessively long and complex genome sequences requires more powerful advanced computational tools. Examples include BioStudio [4], developed for the Sc 2.0 project, and an algorithm employing mixed-integer linear programming to identify non-essential genes for the minimization of the *E. coli* genome [89]. Additionally, a comprehensive computational platform, *DeepCRISPR*, has been developed through deep learning to optimize the design of CRISPR guide RNAs. This platform utilizes a data-driven approach to fully automate the identification of sequences and epigenetic features in the genome that may impact the efficacy of sgRNA knockout. These examples highlight the unique advantages of computational tools and artificial intelligence in the field of synthetic genomics. In terms of cloning steps, with the increase in the DNA scale to be modified, efficient and precise assembly and editing methods have been the focus in recent years. Regarding transfer steps, larger DNA faces lower transfer efficiency. These existing technological constraints unavoidably restrict the scope of research. The transplantation of large DNA offers the potential to overcome the limitations of lacking gene-editing tools in non-model organisms, expanding the host range for manipulating large DNA. Currently, successful transplants of the Mb-level genome have been achieved. Although with specific limitations pertaining to target strains, there is a trend toward expanding the type of host cell. However, it is worth noting that this transfer method still faces challenges related to efficiency. In vivo transfers, such as cell fusion, conjugation transfer, etc., avoid the inefficiencies caused by large DNA extraction. Spontaneous biological pathways, like yeast mating, are often naturally efficient. These directions may be potential strategies for addressing the issue of low transfer efficiency in the future. With the progress in the design, cloning, transfer, and stabilization of large DNA, we are getting closer to comprehensively unraveling the information within the human genome.

## Figures and Tables

**Table 1:** Transfer methods for large DNA.

Type	Recipient	Donor	Method	Size	Refs
Fungi	*S. cerevisiae*		PEG-mediated transformation	1.08 Mb	[17]
	*S. cerevisiae*	*S. cerevisiae*	Yeast mating	Chromosome level	[11,67,68,69]
	*S. cerevisiae*	*S. cerevisiae*	Induced cell fusion	1.03 Mb	[70]
	*S. cerevisiae*	*M. mycoides*	Induced cell fusion	1.8 Mb	[18]
Bacteria	*Agrobacterium*	*F. oxysporum, A. awamori*	*Agrobacterium*-mediated transfer	75 kb	[71]
	*M. capricolum*		PEG-mediated transformation	1.1 Mb	[1]
	*E. coli*		Electroporation	120 kb	[75]
	*E. coli*	*E. coli*	Conjugation	2.3 Mb	[76]
	*B. subtilis*	*B. subtilis*	Conjugation	100 kb	[77]
	*E. coli*	P1 phage	Phage-mediated transfer	100 kb	[78]
